# Feasibility Test of Personalized (N-of-1) Trials for Increasing Middle-Aged and Older Adults’ Physical Activity

**DOI:** 10.21203/rs.3.rs-3788631/v1

**Published:** 2023-12-28

**Authors:** Ciarán P. Friel, Ashley M. Goodwin, Patrick L. Robles, Mark J. Butler, Challace Pahlevan-Ibrekic, Joan Duer-Hefele, Frank Vicari, Samantha Gordon, Thevaa Chandereng, Ying Kuen (Ken) Cheung, Karina W. Davidson

**Affiliations:** Feinstein Institutes for Medical Research; Feinstein Institutes for Medical Research; Feinstein Institutes for Medical Research; Feinstein Institutes for Medical Research; Feinstein Institutes for Medical Research; Feinstein Institutes for Medical Research; Feinstein Institutes for Medical Research; Feinstein Institutes for Medical Research; Feinstein Institutes for Medical Research; Mailman School of Public Health, Columbia University; Feinstein Institutes for Medical Research

**Keywords:** Personalized Trials, N-of-1, Physical Activity, Behavior Change Techniques, Older Adults

## Abstract

**Purpose:**

To test the feasibility of a remotely-delivered intervention to increase low-intensity physical activity (walking) in middle-aged and older adults.

**Design:**

This study used a Personalized (N-of-1) trial design.

**Setting:**

This study took place at a major healthcare system from November 2021 to February 2022.

**Subjects:**

Sixty adults (45–75 years, 92% female, 80% white) were recruited.

**Intervention:**

A 10-week study comprising a 2-week baseline, followed by four 2-week periods where 4 Behavior Change Techniques (BCTs) - self-monitoring, goal setting, action planning and feedback - were delivered one at a time in random order.

**Measures:**

Activity was measured by a Fitbit, and intervention components delivered by email/text. Average daily steps were compared between baseline and intervention. Participants completed satisfaction items derived from the System Usability Scale and reported attitudes and opinions about personalized trials.

**Results:**

Participants rated personalized trial components as feasible and acceptable. Changes in steps between baseline and intervention were not significant, but a large heterogeneity of treatment effects existed, suggesting some participants significantly increased walking while others significantly decreased.

**Conclusions:**

Our intervention was well-accepted but use of BCTs delivered individually did not result in a significant increase in steps. Feasibility and heterogeneity of treatment effects support adopting a personalized trial approach to optimize intervention results.

## Introduction

Physical inactivity is one of the leading causes of preventable death related to behavior in the U.S., and the fourth leading cause of death worldwide.^1,2^ Although the health benefits of physical activity (PA) are well-known, from reducing all-cause mortality and cardiovascular disease risk ^3,4^ to improving mental health,^5^ most Americans fail to meet PA guidelines, with the prevalence of inactivity increasing as people age.^6,7^ This age-related decline in activity often occurs when middle-aged and older adults are already experiencing worsening health, meaning the benefits of PA are particularly potent in this population.^8^ There is also a nonlinear dose–response relationship between PA volume and disease risk reduction, with the greatest decrease in risk seen among insufficiently active adults who increase their PA. This is true even if they remain well below the recommendation of 150 minutes of moderate-to-vigorous PA weekly.^4^ Recent findings have shown that even small increases in PA confer health benefits, with as few as 1000 extra steps per day found to lower the risk of mortality.^4,9^ Despite these seemingly achievable targets, PA behavior change interventions for aging adults have failed to show consistent results.^10^ While there are many possible reasons for this lack of change, two that merit further investigation are the needed type of Behavior Change Techniques (BCTs) used in the intervention (i.e., how many BCTs were used and in what combination), and the heterogeneity of treatment effects across participants (i.e., how many participants increased or decreased their activity, and how many stayed the same).^11^

BCTs are observable, replicable, irreducible intervention components that are thought to be the “active ingredients” of behavior change theories by their influence on the causal processes that direct behavior.^12^ Extensive research has sought to define, classify, and interpret the mounting evidence supporting employment of BCTs to successfully change health behaviors, such as smoking, diet, or PA.^12,13^ From the taxonomy of BCTs established first by Michie et al,^14^ certain BCTs have been identified as active ingredients in increasing PA behavior.^15^ However, there is limited knowledge about the individual effect of these BCTs as they have historically been delivered in various quantities and combinations.^16^ Furthermore, research by Devereux-Fitzgerald et al^17^ proposed that older adults may respond differently to BCT interventions than younger people and lower levels of participation in PA by older adults “suggest that their wants and needs are not being met in the provision or promotion of physical activity” (pg. 15). To address this gap in the literature, this study employed a subset of single BCTs that have been associated with improvements in PA,^15^ focusing on four particularly suited due to their relevance to low-intensity PA (i.e., walking) and ease of remote delivery – self-monitoring, goal setting, action planning and feedback.

The unique needs of middle-aged and older adults may also be lost in the types of trial design traditionally applied - randomized controlled trials (RCTs). RCTs often do not address individual heterogeneity of treatment effects, as participants are randomized to a single treatment, and conclusions only are informative about the hypothetical average participant.^11^ An alternative option, the Personalized (N-of-1) trial design, uses a single-subject, within-subject experimental approach to evaluate the outcomes of different interventions specific to the individual participant.^18,19^ For example, a personalized trial observes one participant over a given time period, delivering randomized exposure to different interventions at scheduled time points, which provides a quantitative estimate of the effects of distinct behavioral interventions on a single participant. Personalized trials have not been widely employed in clinical practice or free-living research designs as delivery of this approach had been perceived as overly burdensome.^20^ However, modern smartphones and wearable sensors have lessened the burden of personalized trial delivery, and enabled wider dissemination of intervention components, while providing highly standardized delivery mechanisms and reducing the manual investment of study personnel. Personalized trials are becoming more commonplace with recent examples including studying fatigue, depression, and back pain.^21–23^

The objective of this study was to assess the feasibility and acceptability of a randomized personalized trial that serially delivered four BCTs, one for each 2-week period, to promote low-intensity PA (walking) in middle-aged and older adults. Wearable technology and smartphone messaging were employed to facilitate completely remote delivery of the study. The study had two primary outcomes (1A & 1B) and one secondary outcome (2A). Outcome 1A was within-person change in daily steps between the baseline and intervention periods, and Outcome 1B was participant satisfaction with personalized trial components. Outcome 2 was assessment of participant attitudes and opinions towards personalized trial components.

## Methods

### Study Population

Employees from a large (> 70,000 employees) healthcare system in the New York area, Northwell Health, were recruited to participate. Recruitment methods included advertisements to those who had previously expressed interest in participating in personalized trials, flyers circulated within the Northwell Health network, and internal social media sites such as Northwell’s employee-only Facebook group (See Appendix A for example). All participants were recruited between November 2021 and February 2022. Employees aged 45–75 years old who reported being healthy, able to walk regularly without health/safety issues and expressed an interest in participating in a personalized trial to increase their walking were eligible to participate. Exclusion criteria included inability to speak/comprehend English, not owning a smart phone capable of receiving text messages and accessing the internet, not having an email address or regular access to an email account, limited mobility and/or have been advised by a clinician not to increase their low-intensity walking, pregnancy, previous diagnosis of a serious mental health condition or psychiatric disorder such as bipolar disorder, or unwillingness to wear an activity tracker (Fitbit) for a minimum of 10 hours a day. Final eligibility was adherence to the study protocol during the baseline period of at least 600 min/day Fitbit wear time and responding to 80% of text messages.

Prior to baseline, consent was presented electronically as an IRB-approved informed consent form requiring a digital signature. The e-consent document contained all elements of informed consent required by applicable federal regulation for the protection of human subjects and elements of authorization required by the HIPAA Privacy Rule. All enrolled participants provided electronic informed consent and received a PDF copy of their electronically signed informed consent form via text message. Electronic consents remain in our secure SharePoint database indefinitely with an IRB-approved data safety monitoring plan in place. The study protocol conforms to the ethical guidelines of the 1975 Declaration of Helsinki as reflected in *a priori* approval by the institution’s human research committee, Northwell Health Institutional Review Board.

### Study Design

**Figure 1:**
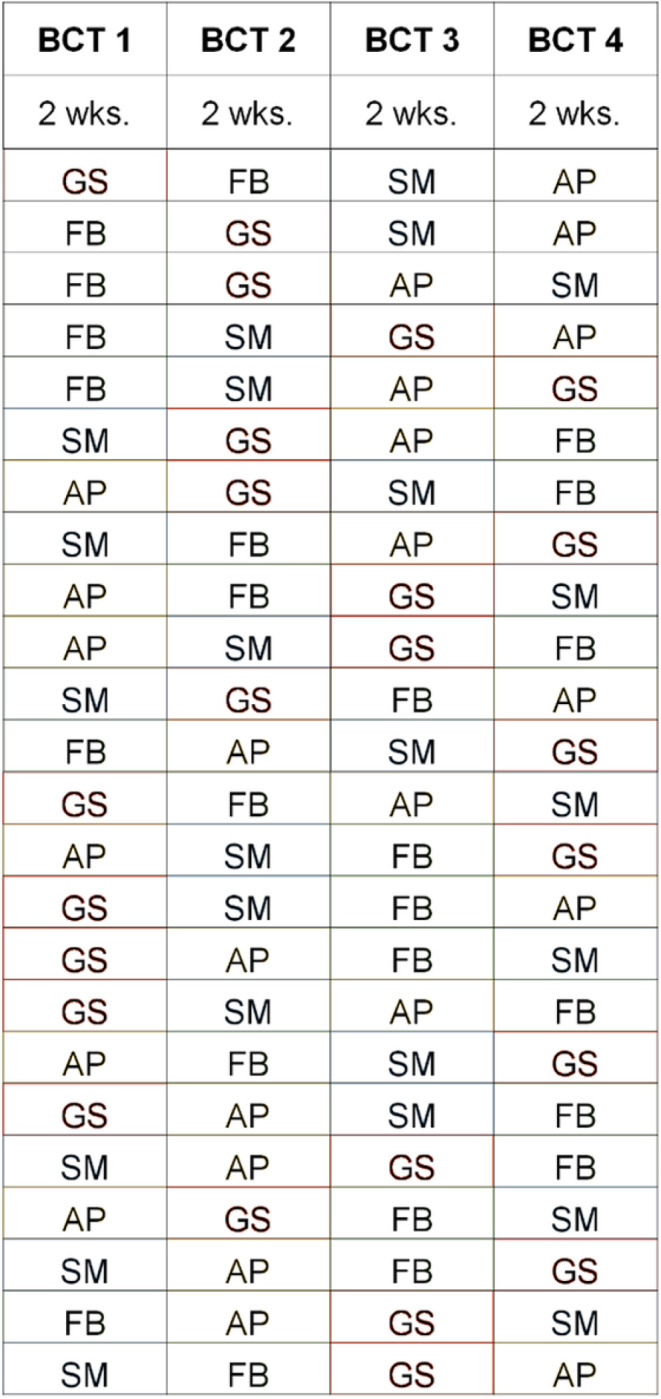
Behavior Change Technique Intervention Sequences GS, goal setting, FB, feedback on behavior, SM, self-monitoring of behavior, AP, action planning

The study had several time periods in its design: baseline period (2 weeks), intervention period (8 weeks), post-intervention follow-up period (at study completion). Each BCT was presented for two weeks during the 8-week active intervention, in a randomly selected order (see Fig. 1). The study statistician assigned the intervention order for each participant.

To optimize retention of participants through all phases, a plan was built into the study’s delivery. When a participant failed to synchronize their device or respond to a survey for two consecutive days, they received an automated text message notifying them that they had dipped below the adherence criteria detailed below. If improvement was not seen in two subsequent days, the clinical research coordinator reached out to the participant by text/phone/email to troubleshoot issues. Retention rates were reported in weekly team meetings to ensure the study was on track for target enrollment (N = 60).

### Onboarding

Once participants consented to participate in the study, but prior to entering the baseline period, they received an “Onboarding Survey” to complete. This form collected demographic and contact information. Once research staff confirmed the participant had completed the required onboarding tasks, an initial study kit was shipped to the participant, including a Fitbit Charge 4^™^ and printed materials to guide the participant through the study. The printed materials explained to participants how to download the Fitbit app to their personal smartphone, set up their study account, how to pair the Fitbit device to their phone, and how to charge/synchronize the Fitbit device. Instructions also included how to navigate the secure messaging platform used to communicate with the study team and answer study related questions/surveys. Anonymous study accounts were created to protect the privacy of the participant so that no identifying information was stored on the Fitbit app.

### Baseline Phase

The baseline period was 2 weeks in duration. During baseline participants were asked to wear their Fitbit all day and night (as tolerated), sync their Fitbit device at least every two days, and to respond to a single daily acknowledgement text message to ensure they were receiving communications (“I have received this text message”). Text messages during baseline and intervention were sent at 9am each morning (Eastern Standard Time). During baseline, participants needed to maintain at least 80% compliance with Fitbit wear time and respond to daily acknowledgement text messages to be eligible for the intervention period. No text message BCT interventions were delivered during baseline as its purpose was to evaluate adherence to the study procedures and provide an average step count for comparison with the intervention periods.

At the end of the baseline period, a clinical research coordinator reviewed individual adherence to Fitbit wear and responsiveness to the daily surveys. Adherence to Fitbit wear was defined as having at least 600 minutes (10 hours) of non-sleep heart rate activity per day. Text message adherence was defined as having responded to the daily acknowledgement messages. Participants who did not achieve at least 80% adherence of both Fitbit wear (≥ 600 minutes) and text message responses during the 2-week baseline period were withdrawn from the study (n = 12). Those that met adherence criteria moved on to the intervention phase.

### Intervention Phase

Participants who successfully completed the baseline were randomized to one of twenty-four sequences of four BCTs. These four BCTs were delivered one at a time, switching every two weeks (See Fig. 1). For example, goal setting messages were sent for two weeks, then self-monitoring prompts for two weeks, and so on for action planning and feedback. On the day before each new two-week period, participants were sent a message describing which BCT was next. BCT messages were automatically delivered daily and incorporated the participant’s own step-count data from baseline and the previous day to generate messages. A description of the BCTs employed and message prompts participants received is provided in Table 1.

### Post-intervention Phase

At the end of the intervention period, participants received a detailed summary of their observed data and a final, comprehensive survey. The personalized summary provided insights about their responses to each of the four BCTs (e.g., “you walked most during the period of time you received the feedback BCT”). In the final survey, participants were asked to rate their satisfaction with the Personalized Trial components using items derived from the System Usability Scale and complete a survey on their attitudes and opinions about the Personalized Trial implementation. These measures are detailed in Appendix B. As compensation, participants could keep their Fitbit, valued at $150, and were given a $100 Paycard for completing all study requirements. Instructions were sent to guide participants on how to unlink the Fitbit device from the study account and set up their own account.

Sample size, all data exclusions, all manipulations, and all measures included in the study were determined a priori. CONSORT guidelines for reporting parallel group randomized trials were followed. The trial was pre-registered on clinicaltrials.gov (NCT04967313). All data, analysis code, and research materials will be made available at the Open Science Framework (OSF).

### Statistical Approach

Assessment measures, treatment repetitions per trial, and maximal trial duration to maintain patient engagement were based on calculations conducted by the study statistician. A trial completion rate > 50% in randomized participants was set a *priori*. Target enrollment (of randomized participants) was determined to be N = 60. Using a 1-sample binomial test at 2.5% significance 1-sided, this target enrollment provided approximately 90% power if the true completion rate were 70% (i.e., 42 participants), thus giving a standard error no greater than 8%. Data were analyzed using R statistical software v 4.2.2 binary for macOS 11 (R Core Team, 2022).

### Primary Outcomes

Within-person change in daily steps. The Fitbit device collected the number of steps taken by each participant throughout the study. Only valid wear days (≥ 600 minutes of non-sleep wear time per day) were included in the analysis. Means and standard deviations for daily steps were calculated for the baseline period and for each BCT intervention period. A linear mixed model with random intercept with autoregressive error of lag 1 (AR [1]) was used to compare the daily step count data between baseline and each BCT intervention period, and the overall intervention. We examined individual treatment effect of each BCT using a linear mixed model with random intercept with AR[1].^24^

Satisfaction with Study Components. At the end of the intervention period, and after receiving their individual study report, participants received a satisfaction survey comprising nine items with a four-point Likert scale (see Appendix B). Each item was preceded by the prompt “How satisfied were you with the following?” and the four-point scale ranged from “Not at all satisfied” (0) to “Very satisfied” (3). Mean and standard deviations for the responses to the items were calculated. The distribution of participant responses for each item assessing satisfaction was calculated and displayed via a bar graph.

### Secondary Outcomes

Participant attitudes and opinions towards personalized trial implementation. To assess attitudes and opinions about personalized trial implementation, participants were asked to respond to an 11-item survey with a 7-point Likert scale (see Appendix C). Each item asked about an aspect of the personalized trial. For example, one item stated, “I enjoyed receiving daily text message prompts and surveys on my cell phone” while another stated “I have found my personalized trial to be very burdensome”. These statements prompted the participant to indicate how much that statement applied to them. The items ranged from “Strongly disagree” (0) to “Strongly agree” (6).” For participant attitudes and opinions about personalized trial components, means and standard deviations for the responses to the items were calculated. To ensure clarity when interpreting the results of this survey, three items were reverse coded from the version participants received (see Appendix C), when graphed. For example, a participant who scored “5, Agree” to original item 1 had their response converted to a score of “1 Disagree” for the revised item (see Supplemental Table S1). The distribution of participant responses for each item assessing attitudes and opinions was entered into a bar graph.

Heterogeneity of Treatment Effect (HTE). As an additional exploratory measure of the utility of conducting an N-of-1 trial for BCT treatment, we examined the heterogeneity of treatment effect (HTE) for the overall intervention.^25^ We first modeled the effects of all BCTs relative to baseline on daily step count using linear mixed models with first-order autocorrelation (AR[1]). We then compared two models of effect of BCTs on steps (one with a random intercept and one with a random slope) using a likelihood ratio test. The random slope model accounts for heterogeneity of treatment effects, whereas the random intercept model does not. If results from the likelihood ratio test show that a random slope model is a better fit than a random intercept model (p-value < 0.05), this would support the interpretation that HTE exists for the effect of a particular intervention on a particular outcome. To further identify whether an N-of-1 trial would be useful for BCT interventions to increase daily step count, we calculated an index of heterogeneity to quantify the HTE of the intervention on step count.^25,26^ This index measures the extent of HTE by incorporating the within-participant variation, between-participant variation, and average treatment effects into the standard deviations of the random slope. Index values range from 0 to1, with scores closer to 1 indicating N-of-1 trials are useful and closer to 0 indicating otherwise. Under special conditions for N-of-1 trials, the index is equal to the statistical power of declaring N-of-1 trials are superior to non-N-of-1 trial alternatives.

### Data Sharing Plan

The trial was pre-registered on clinicaltrials.gov (NCT04967313) and data will be made available on the Open Science Framework (OSF).

## Results

Seventy-two participants entered baseline, of which 60 passed the adherence eligibility criteria. Of those 60 participants, 59 completed the intervention, although intention to treat used data from all 60 participants.

### Participant Characteristics

Sixty participants aged 45–75 (91.67% women) were randomized into the intervention. Table 2 presents participant characteristics. Average steps during baseline were 7225.4 ± 4015.7 (see Supplemental Table S2 and S3 for more detailed wear time and step count data).

### Within-person Step Count

**Figure 2:**
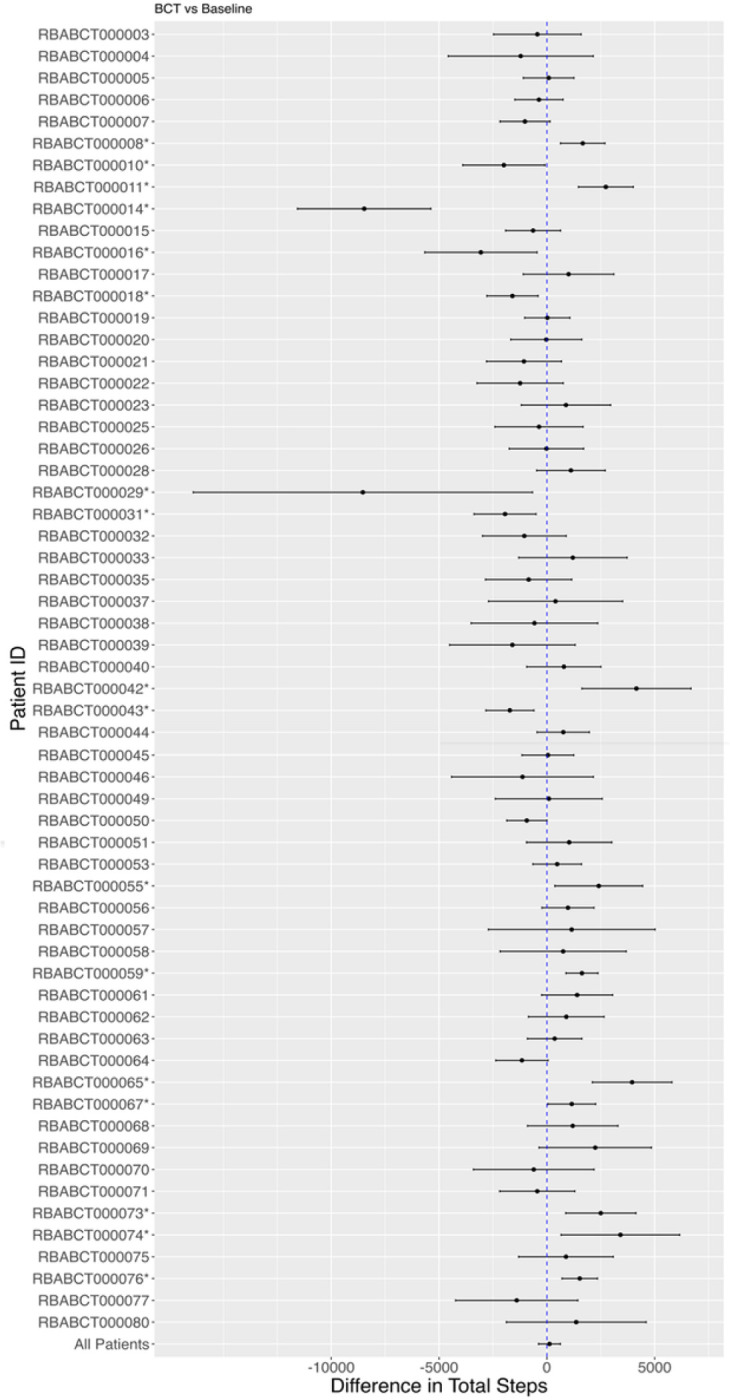
Change in Average Daily Step Counts Between Baseline and Intervention by Participant * Asterisks indicate participants who showed a significant change in steps between baseline and intervention.

Figure 2 provides differences in daily step counts between baseline and the BCT intervention for each individual participant—the appropriate primary analysis for Personalized (N-of-1) trials. Interestingly, and as expected, results show a high degree of heterogeneity wherein some participants had significant and large increases in step counts between baseline and intervention while others showed no change or even a significant decrease.

Supplemental Fig. 2 reports the pooled change in daily step count between baseline and individual BCTs, and baseline vs overall intervention. Our results suggest the overall effect averaged across all participants of the intervention was small and not significant (overall intervention vs baseline: average treatment effect: 120.85, 95% CI (−383.69, 625.40); action planning vs baseline: average treatment effect: 199.25, (−365.80, 764.30), feedback on behavior vs baseline: average treatment effect: 134.84, (−445.16, 714.83), goal setting vs baseline: average treatment effect: −99.35, (−671.78, 473.07), self-monitoring vs baseline: average treatment effect: 225.65, (−344.78, 796.09)).

### Participant Satisfaction with Personalized Trial Components

**Figure 3:**
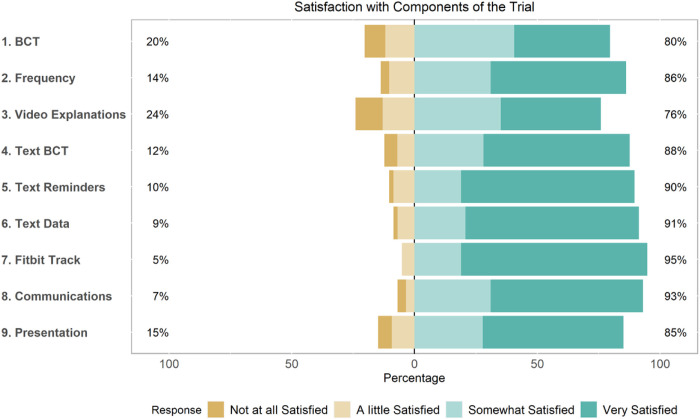
Participant Satisfaction with Personalized Trial Components

Participants reported very high levels of satisfaction with the components of the personalized trial (see Fig. 3). The most highly rated component (95% satisfaction) was use of the Fitbit to track activity. Other highly rated components included study communications and the use of text messaging for data collection (i.e., surveys). Means and standard deviations for all satisfaction survey items can be found in Supplemental Table S1.

### Participant Attitudes and Opinions Towards Personalized Trials Implementation

**Figure 4:**
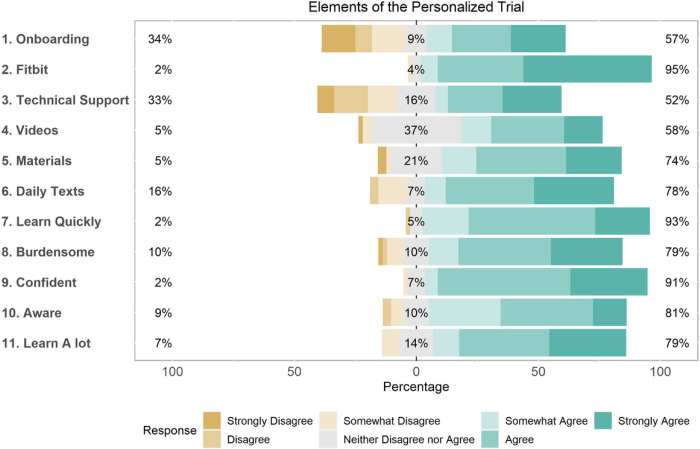
Participant Attitudes and Opinions Towards Personalized Trials

Most participants held positive attitudes and opinions towards the various aspects of personalized trial implementation (see Fig. 4). Onboarding and the initial need for technical support were the two most onerous elements for participants, but once those were completed, everything else was deemed straightforward. Means and standard deviations for the survey questions are detailed in Supplemental Table S1.

### Heterogeneity of Treatment Effect (HTE)

Analyses of the HTE showed that a random slope was a better fit for the data than a random intercept model (Likelihood ratio p-value < 0.001), suggesting that the treatment effects of a BCT intervention on step counts are heterogeneous across different participants. Further, the index of heterogeneity showed that an N-of-1 trial using BCTs to increase step counts would be the most useful relative to a standard approach (Index = 0.802).

## Discussion

This study demonstrated that a Personalized (N-of-1) trial approach conducted using digital delivery for recruitment and intervention was well received by participants and, as expected, delivering individual BCTs serially over an 8-week intervention period significantly increased walking behavior for some adults, significantly decreased it for others, and had no effect for yet others. These findings are noteworthy on three levels. First, to our knowledge, this is the first study to remotely deliver a personalized trial intervention to middle-aged and older adults, and the high adherence to study procedures supports its viability for future use. Second, the individual delivery of key BCTs fills a gap in the literature and provides an important contribution to the body of work on BCTs. Finally, the ability to identify the broad range of effects on individual’s PA by each BCT offers exciting insights into trial design, BCT use and personalization of treatments. These initial findings are consistent with the hypothesis that Personalized (N-of-1) trials may be more useful for identifying BCTs that are efficacious for specific individuals, rather than attempting to find behavioral interventions what work on average, or for the hypothetical ‘average’ middle-aged or older adult.

The high adherence to study procedures and positive feedback from participants demonstrate that a personalized trial approach is feasible for PA interventions in aging adults. While N-of-1 trials have long been considered the ideal of clinical research,^19^ their popularity had been hindered by concerns about technological and methodological limitations. This study, finding high retention and high participant satisfaction scores, showed that continuous data collection using wearable activity trackers, smartphone devices, and automated survey distribution was feasible without excessively burdening the participant or research team.^20^ Thus, a Personalized (N-of-1) trial design, as implemented in this study, is feasible with existing technology and methods to benefit participants. Moreover, establishing a research infrastructure whereby participants can be recruited, onboarded, followed, and off-boarded remotely offers the promise of broader reach and even greater engagement.

Despite a growing body of research on BCTs in PA behavior change,^15^ the ideal dose and blend of BCTs needed to bring about behavior change for the average person is still unclear. Of particular interest in our study was that, despite there not being a significant overall effect, certain individuals did increase their walking, while others decreased or had no change. An exploratory post-hoc statistical power analysis of heterogeneity of treatment effect found only .63 power, suggesting the sample size of 60 was insufficient to detect heterogeneity of individual BCTs in this feasibility Personalized Trial Series. This is useful for considering the next stages of behavioral intervention development needed for this program of research.^27^

The range of step count changes highlight the heterogeneous response to BCTs by individuals. These insights, when considered alongside the broader literature, suggest that even one BCT might be enough to drive behavior change in certain individuals. What the ideal number and type of BCTs is for a given individual warrants additional investigation, in later stage trials as conceptualized by the NIH Stage Model.^28^ Furthermore, some individuals benefited from the intervention while others did not. Such heterogeneity of effect may be undetected with conventional between-subject RCT designs.

The present study found that participants viewed Personalized (N-of-1) trial designs positively and that such designs could be considered feasible. Participants particularly held positive opinions towards wearable activity trackers, remote conduct of the research, and the accessibility of study team members. The findings suggest that participants do not regard Personalized (N-of-1) trials as excessively burdensome or inaccessible. That participants expressed these views towards the study is critical and underscores the newfound feasibility of N-of-1 trials, which rely on low participant burden and ease of conduct.

This study has several limitations. Firstly, only employees of a healthcare system in good health (i.e., ambulatory without limitation, no comorbidities, etc.) were recruited so these findings may not be generalizable to individuals with health complications or those who work outside of a healthcare system. Secondly, while all participants self-reported a desire to increase their walking, average baseline step counts were higher than is generally considered sedentary (< 5,000). While benefits from increased walking exist for those over the 5,000-step threshold,^29^ generalizability to more sedentary individuals could be reduced. Future studies should consider the activity level of their target sample and screen out participants in baseline who exceed this threshold. Finally, while many aspects of the remote delivery were successful, certain technical issues were encountered, such as delayed delivery of messages to participants. Future studies should ensure robust pre-trial testing takes place and a regular, scheduled quality control protocol put in place.

## Conclusion

This remotely delivered study contributes broadly to the science of PA behavior change in middle-aged and older adults and offers exciting direction to future research. BCTs are seen as key components of behavior change, but their individual influence is not well understood. The personalized trial design used in this study allowed for unprecedented examination of a BCT intervention at the single subject level. This approach allows an investigation of the heterogeneity of effect observed; some participants may have benefited from receiving a given BCT, whereas others did not. Our study’s findings demonstrate the novel scientific insights attainable when a Personalized (N-of-1) trial approach is employed. Lastly, this study offers a vision of conducting future, later NIH stage model informed Personalized (N-of-1) trials where information derived from the user’s own physiological data, rather than generic guidelines, are used to inform the prescription for each person and provide appropriate behavioral interventions that speak to every individual.

## Figures and Tables

**Table 1:** Description of Behavior Change Technique Messages

BCT	Modality	Text Message Prompt
Goal Setting	Text message delivered daily to encourage the participant to set a goal of exceeding their average daily step count from baseline. The baseline average step count was included in the message.	“Good morning [participant name]. Is your goal today to walk an extra 2,000 steps more than your baseline average (#### total steps)?”
Action Planning	Text message delivered each morning encouraging the participant to plan for an additional walking bout. The baseline average step count was included in the message.	“Good morning [participant name]. Take a minute and plan for today how, where, and when you can walk an extra 2,000 steps over your baseline average (#### total steps).”
Self-Monitoring of Behavior	Ongoing self-monitoring is prompted by a daily morning text message. The baseline average step count was included in the message.	“Good morning [participant name]. Check your Fitbit dashboard for yesterday. Did you take an extra 2,000 steps more than your baseline average (#### total steps)?”
Feedback on Behavior	Text message delivered each morning stating whether participant’s goal had been met on the day prior. The baseline average step count was included in the message.	“Good morning [participant name]. You met your goal yesterday by walking at least an extra 2,000 steps more than your baseline average (you walked #### steps total and your goal was ####).” OR “Good morning [participant name]. You did not meet your goal yesterday by walking at least an extra 2,000 steps more than your baseline average (you walked ### steps total, and your goal was ####)

**Table 2:** Participant Characteristics at Baseline

Variable	Total Sample N = 60
***Age Range (years)***	
45–55	31
56–65	27
66–75	2
**Sex**	91.67%
Female	
**Ethnicity**	
Hispanic	5%
Non-Hispanic	95%
**Race**	
Black or African American	8%
White	80%
American Indian or Alaska Native	2%
Asian	7%
Unknown or Not Reported	3%
**Health**	
BMI (kg/m^2^)	29.57 + 5.62

Data presented as % sample or mean + SD.

## References

[1] Kohl HW, Craig CL, Lambert EV, Inoue S, Alkandari JR, Leetongin G, The pandemic of physical inactivity: global action for public health. Lancet. 2012;380(9838):294–305. 22818941 10.1016/S0140-6736(12)60898-8

[2] Mokdad AH, Marks JS, Stroup DF, Gerberding JL. Correction: actual causes of death in the United States, 2000. Jama. 2005;293(3):293–294. 15657315 10.1001/jama.293.3.293

[3] Piercy KL, Troiano RP, Ballard RM, Carlson SA, Fulton JE, Galuska DA, The physical activity guidelines for Americans. Jama. 2018;320(19):2020–2028. 30418471 10.1001/jama.2018.14854 PMC9582631

[4] Cunningham C, O’Sullivan R, Caserotti P, Tully MA. Consequences of physical inactivity in older adults: A systematic review of reviews and meta-analyses. Scand J Med Sci Sports. 2020;30(5):816–827. 32020713 10.1111/sms.13616

[5] Callow DD, Arnold-Nedimala NA, Jordan LS, Pena GS, Won J, Woodard JL, The mental health benefits of physical activity in older adults survive the COVID-19 pandemic. Am J Geriatr Psychiatry. 2020;28(10):1046–1057. 32713754 10.1016/j.jagp.2020.06.024 PMC7831892

[6] Diaz KM, Howard VJ, Hutto B, Colabianchi N, Vena JE, Safford MM, Patterns of sedentary behavior and mortality in US middle-aged and older adults: a national cohort study. Ann Intern Med. 2017;167(7):465–475. 28892811 10.7326/M17-0212 PMC5961729

[7] Du Y, Liu B, Sun Y, Snetselaar LG, Wallace RB, Bao W. Trends in adherence to the physical activity guidelines for Americans for aerobic activity and time spent on sedentary behavior among US adults, 2007 to 2016. JAMA Netw Open. 2019;2(7):e197597–e197597. 31348504 10.1001/jamanetworkopen.2019.7597 PMC6661709

[8] Langhammer B, Bergland A, Rydwik E. The importance of physical activity exercise among older people. BioMed Res Int. 2018. 10.1155/2018/7856823 PMC6304477 30627571

[9] Hall KS, Hyde ET, Bassett DR, Carlson SA, Carnethon MR, Ekelund U, Systematic review of the prospective association of daily step counts with risk of mortality, cardiovascular disease, and dysglycemia. Int J Behav Nutr Phys Act. 2020;17(1):1–14. 32563261 10.1186/s12966-020-00978-9 PMC7305604

[10] Stockwell S, Schofield P, Fisher A, Firth J, Jackson SE, Stubbs B, Digital behavior change interventions to promote physical activity and/or reduce sedentary behavior in older adults: A systematic review and meta-analysis. Exp Gerontol. 2019;120:68–87. 30836130 10.1016/j.exger.2019.02.020

[11] Greenfield S, Kravitz R, Duan N, Kaplan SH. Heterogeneity of treatment effects: implications for guidelines, payment, and quality assessment. Am J Med. 2007;120(4):S3–S9. 10.1016/j.amjmed.2007.02.002 17403380

[12] Michie S, Richardson M, Johnston M, Abraham C, Francis J, Hardeman W, The behavior change technique taxonomy (v1) of 93 hierarchically clustered techniques: building an international consensus for the reporting of behavior change interventions. Ann Behav Med. 2013;46(1):81–95. 23512568 10.1007/s12160-013-9486-6

[13] Michie S, Johnston M. Theories and techniques of behaviour change: Developing a cumulative science of behaviour change. Health Psychol Rev. 2012;6(1):1–6.

[14] Michie S, Ashford S, Sniehotta FF, Dombrowski SU, Bishop A, French DP. A refined taxonomy of behaviour change techniques to help people change their physical activity and healthy eating behaviours: the CALO-RE taxonomy. Psychol Health. 2011;26(11):1479–1498. 21678185 10.1080/08870446.2010.540664

[15] French DP, Olander EK, Chisholm A, McSharry J. Which behaviour change techniques are most effective at increasing older adults’ self-efficacy and physical activity behaviour? Ann Behav Med. 2014;48(2):225–234. 24648017 10.1007/s12160-014-9593-z

[16] Samdal GB, Eide GE, Barth T, Williams G, Meland E. Effective behaviour change techniques for physical activity and healthy eating in overweight and obese adults; systematic review and meta-regression analyses. Int J Behav Nutr Phys Act. 2017;14:1–14. 28351367 10.1186/s12966-017-0494-y PMC5370453

[17] Devereux-Fitzgerald A, Powell R, Dewhurst A, French DP. The acceptability of physical activity interventions to older adults: A systematic review and meta-synthesis. Soc Sci Med. 2016;158:14–23. 27104307 10.1016/j.socscimed.2016.04.006

[18] Davidson KW, Silverstein M, Cheung K, Paluch RA, Epstein LH. Experimental designs to optimize treatments for individuals: personalized N-of-1 trials. JAMA Pediatr. 2021;175(4):404–409. 33587109 10.1001/jamapediatrics.2020.5801 PMC8351788

[19] Guyatt G, Sackett D, Taylor DW, Ghong J, Roberts R, Pugsley S. Determining optimal therapy— randomized trials in individual patients. N Engl J Med. 1986;314(14):889–892. 2936958 10.1056/NEJM198604033141406

[20] Davidson KW, Peacock J, Kronish IM, Edmondson D. Personalizing behavioral interventions through single-patient (N-of-1) trials. Soc Personal Psychol Compass. 2014;8(8):408–421. 25267928 10.1111/spc3.12121 PMC4175746

[21] Butler M, D’Angelo S, Lewis C, Miller D, Perrin A, Suls J, Series of virtual light therapy interventions for fatigue: a feasibility pilot study protocol for a series of personalized (N-of-1) trials. BMJ Open. 2022;12(10):e055518. 10.1136/bmjopen-2021-055518 PMC9608534 36283748

[22] Butler Mark “A Series of Virtual Interventions for Chronic Lower Back Pain: A Feasibility Pilot Study for a Series of Personalized (N-of-1) Trials.” Harvard data science review vol. 4,SI3. 2022; 10.1162/99608f92.72cd8432. PMC10443938 37609556

[23] D’Angelo S, Ahn H, Miller D, Monane R, Butler M. Personalized feedback for personalized trials: construction of summary reports for participants in a series of personalized trials for chronic lower back pain. Harvard data science review, Special Issue 3. 2022; 10.1162/99608f92.d5b57784 PMC10673635 38009134

[24] Chandereng T, Liao Z, D’Angelo S, Butler M, Davidson KW, Cheung YK. Role of Digital Healthcare Approaches in the Analysis of Personalized (N-of-1) Trials. In: Personal Health Informatics: Patient Participation in Precision Health. Cham: Springer International Publishing; 2022. p. 131–146.

[25] Cheung K, Mitsumoto H. Evaluating personalized (N-of-1) trials in rare diseases: How much experimentation is enough. Harvard Data Science Review. (Special Issue 3). 2022 doi:10.1162/99608f92.e11adff0. PMC10813653 38283317

[26] Cheung YK. Personalized (N-of-1) trial design tools. [Internet]. 2023 [cited 2023 Mar 1]. Available from: https://roadmap2health.io/cmi/

[27] Onken LS. History and evolution of the NIH stage model. In: Evidence–based practice in action: bridging clinical science and intervention; 2019. p. 28–42.

[28] Onken LS, Carroll KM, Shoham V, Cuthbert BN, Riddle M. Reenvisioning clinical science: unifying the discipline to improve the public health. Clin Psychol Sci. 2014;2(1):22–34. 25821658 10.1177/2167702613497932 PMC4374633

[29] Banach M, Lewek J, Surma S, Penson PE, Sahebkar A, Martin SS, The association between daily step count and all-cause and cardiovascular mortality: a meta-analysis. Eur J Prev Cardiol. 2023. doi:10.1093/eurjpc/zwad229. Epub ahead of print. 37555441

